# Development of a job satisfaction measure for clinical research professionals: A mixed methods approach

**DOI:** 10.1017/cts.2025.34

**Published:** 2025-03-24

**Authors:** Jacqueline M. Knapke, John Kues, Spencer K. Harris, Denise C. Snyder, Stephanie A. Freel, Harini Pallerla, Jessica Fritter, Angela Mendell, Carolynn T. Jones

**Affiliations:** 1 Center for Clinical and Translational Science and Training, University of Cincinnati, Cincinnati, OH, USA; 2 Division of Gastroenterology, Hepatology and Nutrition, The Ohio State University Wexner Medical Center, Columbus, OH, USA; 3 Office of Clinical Research, Clinical Translational Science Institute, School of Medicine, Duke University, Durham, NC, USA; 4 Department of Family and Community Medicine, University of Cincinnati, Cincinnati, OH, USA; 5 College of Nursing, College of Medicine, Center for Clinical Translational Science, The Ohio State University, Columbus, OH, USA

**Keywords:** Clinical research professional, job satisfaction, retention, workforce development, factor analysis, principal components analysis

## Abstract

**Background:**

Clinical research professionals (CRPs) are essential members of research teams serving in multiple job roles. However, recent turnover rates have reached crisis proportions, negatively impacting clinical trial metrics. Gaining an understanding of job satisfaction factors among CRPs working at academic medical centers (AMCs) can provide insights into retention efforts.

**Materials/Methods:**

A survey instrument was developed to measure key factors related to CRP job satisfaction and retention. The survey included 47 rating items in addition to demographic questions. An open-text question solicited respondents to provide their top three factors for job satisfaction. The survey was distributed through listservs of three large AMCs. Here, we present a factor analysis of the instrument and quantitative and qualitative results of the subsequent survey.

**Results:**

A total of 484 CRPs responded to the survey. A principal components analysis with Varimax rotation was performed on the 47 rating items. The analysis resulted in seven key factors and the survey instrument was reduced to 25 rating items. Self-efficacy and pride in work were top ranked in the quantitative results; work complexity and stress and salary and benefits were top ranked in the qualitative findings. Opportunities for education and professional development were also themes in the qualitative data.

**Discussion:**

This study addresses the need for a tool to measure job satisfaction of CRPs. This tool may be useful for additional validation studies and research to measure the effectiveness of improvement initiatives to address CRP job satisfaction and retention.

## Introduction

Clinical research is an indisputable cornerstone in pursuing public health, providing invaluable contributions and yielding substantial benefits [1]. The increasing prevalence of clinical trials and human participatory research reflects a collective commitment to driving innovation, bridging the gap between scientific discovery and clinical application and addressing pressing healthcare challenges. Clinical research professionals (CRPs) are essential for successful clinical studies at the research site, ensuring adherence to regulations and ethical considerations [2–4]. CRP job satisfaction and retention directly impact research quality and efficiency. Satisfied healthcare professionals tend to be motivated, leading to improved participant experiences and outcomes [5]. Retention of these valuable employees fosters a cumulative knowledge base, promotes innovation, and reduces turnover costs [6].

Historically, the clinical research field has grappled with high CRP turnover rates and a competitive job market, leading to a “war for talent” that jeopardizes the quality of clinical trials and patient outcomes [7]. In response, a variety of strategies have been employed, ranging from enhancing career progression pathways and increasing salaries, to fostering stronger collegial connections and making strategic decisions around staffing, such as utilizing contractors or reshaping existing roles [8–10]. Despite these efforts, there remains a pervasive belief among employees that the most effective strategy for meeting their professional needs is to seek opportunities outside their current organization [9,11].

Academic medical centers (AMCs) face unique challenges to retaining competent CRPs for complex reasons, including limited funding to provide training and professional development initiatives, lack of transparent avenues for promotion, and feeling under-appreciated and burned out [8,12,13]. Existing CRP workforce challenges were worsened by COVID-19; one study found that 37% of AMCs reported decreased staffing and increased turnover as a result of the pandemic [7]. Retention rates of CRPs in AMCs are not well-defined, but in clinical research organizations (CROs), the average turnover rate from 2017 to 2021 was 26.2%, and one AMC reported in 2022 turnover rates between 18.7% and 37.5% [9,14]. Moreover, turnover rates among staff working in oncology clinical research have been especially high [15,16].

Job satisfaction is a critical yet understudied component that could be used to improve the retention of highly competent CRPs at AMCs. Studies of job satisfaction in this population are minimal and dated [17,18]. Job satisfaction surveys are commonly used in other fields, including healthcare, business, and education sectors [19–21], elucidating its often complex and multifactorial nature, encompassing factors both intrinsic and extrinsic to an individual and an institution [22–24]. Enhancing job satisfaction and retention rates among CRPs requires a standardized means for measuring the outcome of myriad retention interventions. The postpandemic landscape of increased CRP turnover demands a re-examining of factors influencing CRP job satisfaction. Herein, we describe a factor analysis conducted to develop an iterative survey tool to identify and measure crucial CRP job satisfaction factors, including a report of results from initial use of the survey. The instrument described in this manuscript will provide a critical tool for assessing interventions designed to improve the workforce landscape.

## Methods

### Survey and study development

A working group consisting of CRPs, managers, and researchers from three large AMCs (The Ohio State University, University of Cincinnati, and Duke University) was formed to design and launch an online survey to better understand factors associated with CRP job satisfaction and develop a tool for future research to evaluate CRP job satisfaction. The survey instrument was informed by published CRP surveys[3] and job satisfaction survey items published by the Society for Human Resource Management (SHRM) [25], and several guidelines described by Burns, et. al. (2008) were followed in the survey design [26]. We used 5-point Likert scales to measure factors that are critical to CRP job satisfaction and retention: *How important to you is…*(1= Not at all important to 5 = Extremely important), *and How appreciated do you feel…*(1 = Not at all appreciated to 5 = Extremely appreciated). We also asked several questions that solicited the respondent’s level of agreement: (1 = Strongly disagree, 2 = Somewhat disagree, 3 = Neither agree nor disagree, 4 = Somewhat agree, 5 = Strongly agree). The agreement questions included statements about the work environment, onboarding/training, team dynamics, and recognition. The original survey included 47 rating items. The rating items were grouped into three questions: 1) How important to your job satisfaction are the following factors (8 items), 2) Indicate how appreciated you feel by the following groups of people (6 items), 3) Indicate your level of agreement with the following statements related to (a) work/task-related questions (10 items), (b) work environment (9 items), (c) safety and equity (6 items), (d) training and personal fulfillment (8 items). In addition to soliciting demographic data, a final open-ended question was included: “Please list three things on your “wish list” that would make your role in clinical research more satisfying” to gain additional insights on study results and ensure we were capturing factors identified as important by CRP respondents. The project was determined exempt by the Institutional Review Boards of the three participating institutions, and the instrument was administered via Qualtrics^TM^ (Provo, UT). A cover letter was included with the Qualtrics link describing the project. Clinical research operational leaders were engaged at all institutions to help with recruitment efforts.

### Population

Eligibility criteria required that the participants be classified as nonfaculty CRPs employed to support the operation of clinical research studies. Employing a convenience sampling strategy, the survey URL was distributed to CRP listservs at each of the three institutions. These distribution lists included 2,127 individuals. The survey was available for 10 weeks, from late November 2022 to early February 2023, and included two reminders.

### Statistical methods

#### Factor analysis

The 47 rating questions were initially included in a principal component analysis (PCA) that also included a Varimax rotation. A PCA provides a tool to explore the survey data, preserves variability, and helps to define the dimensions of the data; Varimax rotation is designed to maximize the independence among factors [27]. The factors from this initial analysis were used to reduce the number of items from 47 to 25 based on a factor loading cutoff of 0.500 and discussion among the investigators. The final analysis identified seven factors. Standard scores for each of the factors were generated by dividing the total factor score by the number of items for each factor. A total Job Satisfaction Index score was calculated by summing the scores of the 25 items. A standardized Job Satisfaction Index score was calculated by adding the standardized factor scores across the seven factors. PCAs were performed using IBM SPSSv29.0^©^.

#### Quantitative and qualitative methods

Quantitative survey data were decoupled from qualitative survey data and analyzed separately. Survey responses from the quantitative survey items were analyzed in Excel using descriptive statistics. Incomplete responses were included in all analyses. For the qualitative survey analysis, we employed a phenomenological approach in order to describe the lived experiences of CRPs and the meaning assigned to those experiences. Survey questions from the qualitative survey items were analyzed using content analysis to describe key factors influencing CRP job satisfaction using frequencies [28]. One primary coder (JK), who has doctoral-level training in qualitative methods and 15 years of experience in research workforce development, manually coded qualitative data in Excel, bringing initial codes and interpretive questions to the research team for discussion and resolution. Qualitative themes were intentionally aligned with factors identified in the quantitative analysis, although all data were coded whether they fit within a factor or not, allowing for the identification of new themes not represented in the quantitative data. All results were then reviewed and discussed by the research team so that quantitative and qualitative results could be compared and contrasted. The team met twice a month over 6 months to complete the PCA with Varimax rotation as well as the qualitative data analysis. Methods and results were discussed regularly in order to interpret findings appropriately and reach an agreement on the final results.

## Results

### Population data

A total of 484 (22.8% response rate) CRPs responded to the survey. Of those, 86% were female, 93% identified as non-Hispanic, and 81% identified as white. The largest percentage of respondents (49%) reported their highest level of education being a baccalaureate degree, and the median annual salary range was between $65,000 and $75,000. Most respondents (53%) reported their current role as clinical research coordinators or clinical research nurses, and 21% reported being clinical research managers or directors. There was nearly equal distribution across experience levels: 24% of the respondents had been employed in clinical research for 2–5 years, 24% had been employed in clinical research for > 5 to 10 years, and 30% for over 10 years, respectively. Almost one-third of the respondents (31%) had worked in their current team for 2–5 years. Finally, over a quarter (27%) had their current job titles for 2–5 years and 15% for 5–10 years. Table 1 provides complete demographic information for survey respondents.

**Table 1.** Summary of demographic characteristics of survey respondents (*N* = 484)

Survey Item	n (%)
**Age**	
18-25	56 (12)
26-35	165 (34)
36-45	101 (21)
46-55	87 (18)
>55	72 (15)
N/A	3 (1)
**Gender**	
Female	418 (86)
Male	54 (11)
Nonbinary	3 (1)
Prefer not to say or N/A	9 (2)
**Race**	
American Indian or Alaska Native	2 (0)
Asian	21 (4)
Black or African American	35 (7)
More than one race	17 (4)
Other	17 (4)
White	392 (81)
**Ethnicity**	
Non-Hispanic	451 (93)
Hispanic or Latino	30 (6)
N/A	3 (1)
**Highest Degree**	
Doctoral	42 (9)
Master’s	165 (34)
Bachelor’s	238 (49)
Associate’s	20 (4)
Some college	15 (3)
High school	6 (1)
**Current Salary Range**	
<$25,000	3 (1)
$25,000–$35,000	9 (2)
$35,000–$45,000	47 (10)
$45,000–$55,000	70 (14)
$55,000–$65,000	77 (16)
$65,000–$75,000	100 (21)
$75,000–$85,000	58 (12)
$85,000–$95,000	46 (10)
$95,000–$105,000	30 (6)
>$105,000	38 (8)
N/A	6 (1)
**Salaried or Hourly**	
Salaried	370 (76)
Hourly	114 (24)
**Employment**	
Full-time	470 (97)
Part-time	14 (3)
Certification Status*	
Yes	150 (31)
No	333 (69)
N/A	1 (0)
**Length of Employment in CTR**	
<1 year	51 (11)
1–2 years	58 (12)
2–5 years	116 (24)
5–10 years	117 (24)
10–15 years	51 (11)
>15 years	90 (19)
N/A	1 (0)
**Job Category**	
Clinical Research Coordinator	204 (42)
Clinical Research Manager/Director	102 (21)
Clinical Research Nurse	51 (11)
Other/NA	47 (10)
Clinical Research Assistant	29 (6)
Regulatory Affairs	30 (6)
Compliance/Quality Assurance	10 (2)
Data Entry/Data Management	10 (2)
Lab or Phlebotomy Technician	1 (0)

*e.g., Certified by a professional organization such as the Association for Clinical Research Professionals (ACRP), the Society of Clinical Research Associates (SOCRA), the International Association of Clinical Research Nurses (IACRN), Regulatory Affairs Professionals Society (RAPS), or Public Responsibility in Medicine & Research (PRIM&R).

### Factor analysis

The initial PCA with Varimax rotation was performed on the 47 rating items. It resulted in 11 factors that explained 63.13% of the variance. We removed items with factor loadings of less than 0.500 after the investigators reviewed the factor analysis findings. Sixteen items were removed based on this cutoff criterion. We recalculated the PCA with Varimax rotation on the remaining 31 items. This resulted in nine factors that explained 65.73% of the variance. Two items were removed based on factor loadings of less than 0.500. The remaining 29 items were reviewed by the investigator team. Four additional items were removed either because they were highly correlated with one or more items in their factor (2 items) or because there was some concern about possible misinterpretation of the question by the respondents (2 items).

The final 25 items were reanalyzed using PCA and Varimax rotation. The analysis produced seven factors, with each factor including two to seven items. The total variance explained by the seven factors was 64.33%. The factors each contained between two and seven items (Table 2). The standardized mean scores across the factors ranged from 3.54 (Factors 1 and 6) to 4.48 (Factor 7). No items had factor loadings lower than 0.597 (Table 3). The mean total Standardized Job Satisfaction Index score was 28.35 (SD = 2.64) with a range of 17.00 to 34.71. The scores were approximately normally distributed with a slight skew to the left (lower scores). Supplement 1 provides the final survey instrument.

**Table 2.** Means and standard deviations for standardized factors (*N* = 471)

FACTOR	n of Items	Standardized	Standard Deviation
Factor 1: Appreciation and workplace equity	7	3.54	0.88
Factor 2: Team	4	4.10	0.78
Factor 3: Pride in work and advancement	4	4.14	0.74
Factor 4: Work environment	3	4.31	0.63
Factor 5: Salary and benefits	3	4.24	0.65
Factor 6: Work complexity and stress	2	3.54	0.96
Factor 7: Work self-efficacy	2	4.48	0.67
Total	25	28.35	2.64

**Table 3.** Job satisfaction index factors

FACTOR 1: APPRECIATION AND WORKPLACE EQUITY (15.98% Variance Explained)	
Item	Factor Loading
Please indicate how appreciated you feel by the following groups of people? Your supervisor	0.751
Please indicate your level of agreement with each of the following statements. My supervisor treats all members of the team equally	0.751
Please indicate your level of agreement with each of the following statements. My opinions are valued by my supervisor	0.743
Please indicate your level of agreement with each of the following statements. There is an equal opportunity for promotion in my department	0.694
Please indicate how appreciated you feel by the following groups of people? Your organization (institution)	0.597
Please indicate how appreciated you feel by the following groups of people? Your department	0.685
Please indicate your level of agreement with each of the following statements. I feel informed about upcoming changes in my organization	0.652
**FACTOR 2: TEAM** (10.98% Variance Explained)	
Please indicate your level of agreement with each of the following statements: The people I work with cooperate to get the job done	0.769
Please indicate your level of agreement with each of the following statements: There is cohesion in my team- we are a real team	0.790
Please indicate how appreciated you feel by the following groups of people: Your team	0.675
Please indicate your level of agreement with each of the following statements: I feel a sense of belonging in my work group	0.609
**FACTOR 3: PRIDE IN WORK AND ADVANCEMENT** (10.87% Variance Explained)	
Please indicate your level of agreement with each of the following statements: My work makes a positive impact on current and future patient care	0.758
Please indicate your level of agreement with each of the following statements: My work gives me a feeling of personal accomplishment	0.739
Please indicate your level of agreement with each of the following statements: Academic education in clinical research can improve my job progression	0.616
Please indicate your level of agreement with each of the following statements: I am proud to work for my organization	0.685
**FACTOR 4: WORK ENVIRONMENT** (7.79% Variance Explained)	
How important to your job satisfaction are the following factors: Coworkers	0.800
How important to your job satisfaction are the following factors: Working Conditions	0.763
How important to your job satisfaction are the following factors: The work itself	0.742
**FACTOR 5: SALARY AND BENEFITS** (7.06% Variance Explained)	
How important to your job satisfaction are the following factors: Salary	0.823
How important to your job satisfaction are the following factors: Promotion Outlook	0.769
How important to your job satisfaction are the following factors: Benefits	0.628
**FACTOR 6: WORK COMPLEXITY AND STRESS** (5.85% Variance Explained)	
Please indicate your level of agreement with each of the following statements: My study tasks are difficult and complex	0.784
Please indicate your level of agreement with each of the following statements: I work in a very stressful environment	0.733
**FACTOR 7: WORK SELF-EFFICACY** (5.83% Variance Explained)	
Please indicate your level of agreement with each of the following statements: I feel confident in completing my study tasks	0.793
Please indicate your level of agreement with each of the following statements: My work product is high caliber (quality)	0.769

The possible range of raw index scores was 25 to 125 (values of 1 to 5 on each item × 25 items). The range of actual raw scores was 51–123. The raw quartile scores were 93 (25^th^ percentile), 102 (50th percentile), and 109 (75th percentile).

A Kaiser–Meyer–Olkin test indicated that the sample was adequate for the factor analysis that was performed (KMO = .858; >0.600 is considered good). Additionally, Bartlett’s test of sphericity found that the underlying correlation matrix was unrelated to an identity matrix (approximate Chi-Square = 4415.438; df = 300, *p* < .001) indicating that the factor analysis was appropriate to perform. Cronbach’s alphas were calculated on each of the seven factors (Table 4). Values ranged from 0.9 to 0.5. Factors with larger numbers of items (factors 1–3) had values of 0.8–0.9 while factors with less than four items (factors 4–7) had somewhat lower values as might be expected (0.5–0.7).

**Table 4.** Cronbach’s alpha values for each factor

FACTOR	Cronbach’s Alpha	n of Items
Factor 1: Appreciation and workplace equity	0.9	7
Factor 2: Team	0.8	4
Factor 3: Pride in work and advancement	0.8	4
Factor 4: Work environment	0.5	3
Factor 5: Salary and benefits	0.7	3
Factor 6: Work complexity and stress	0.6	2
Factor 7: Work self-efficacy	0.5	2

To test the validity of the final Job Satisfaction Index, a Split Half analysis was conducted. A random draw of half of the survey responses was used for the analysis. It resulted in 7 factors with a distribution of the 25 items in the same factors as the original Index. It explained 65.57% of the variance with a KMO of 0.847 and a Bartlett’s analysis value of 2341.436; df (300), *p* < .001.

### Descriptive results

**Figure 1.**
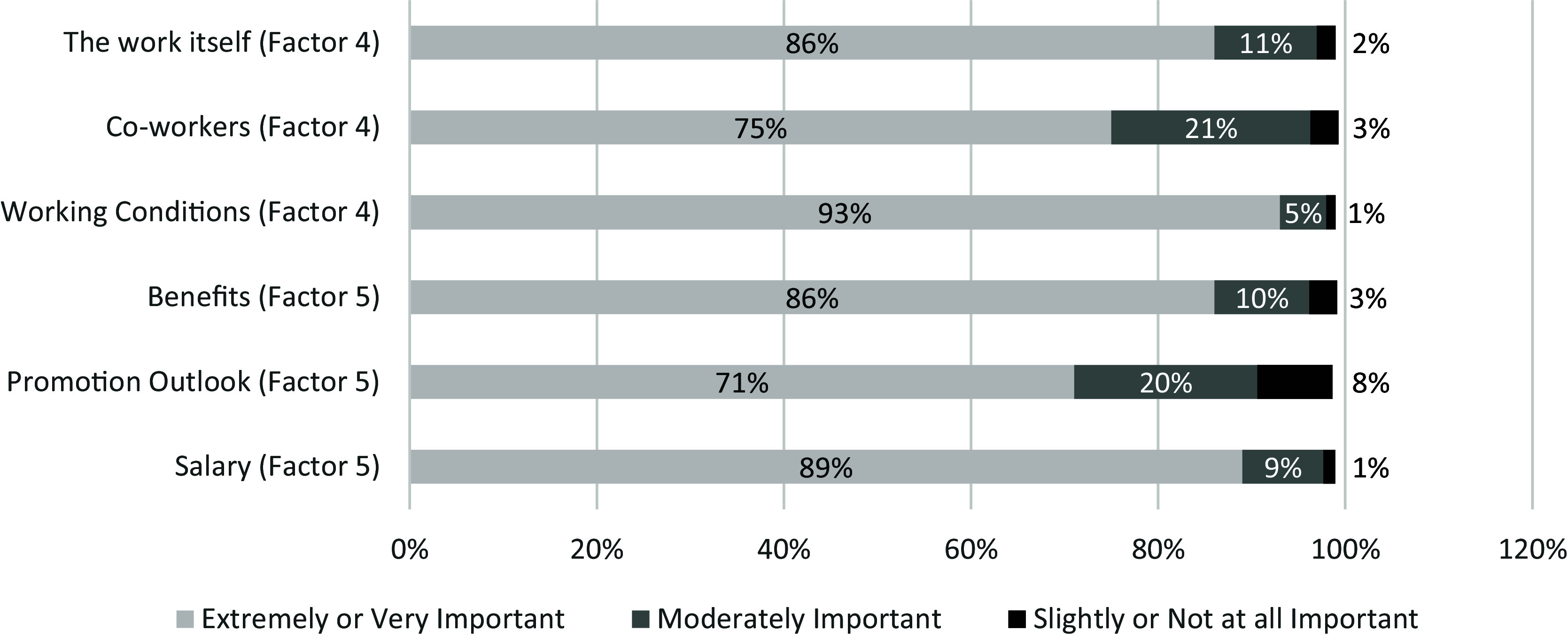
Results of “How important is each of the following items to your job satisfaction?” (*N* = 484).

**Figure 2.**
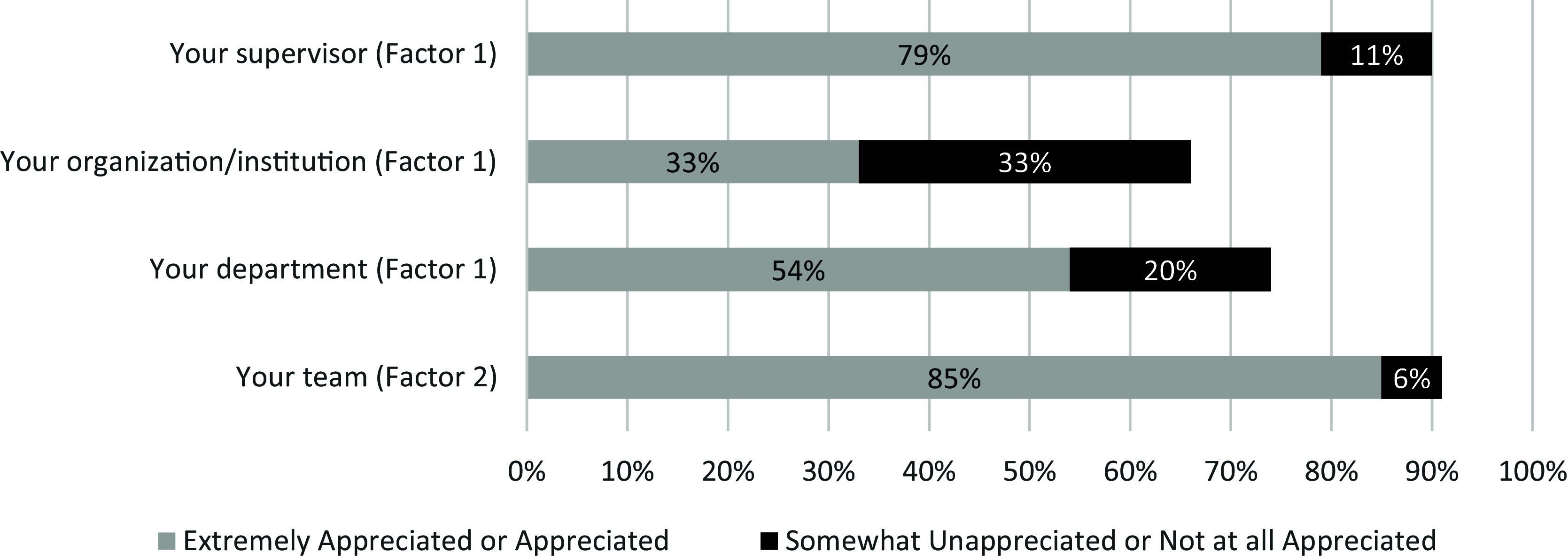
Results of “Indicate how appreciated you feel by:” (*N* = 484).

**Figure 3.**
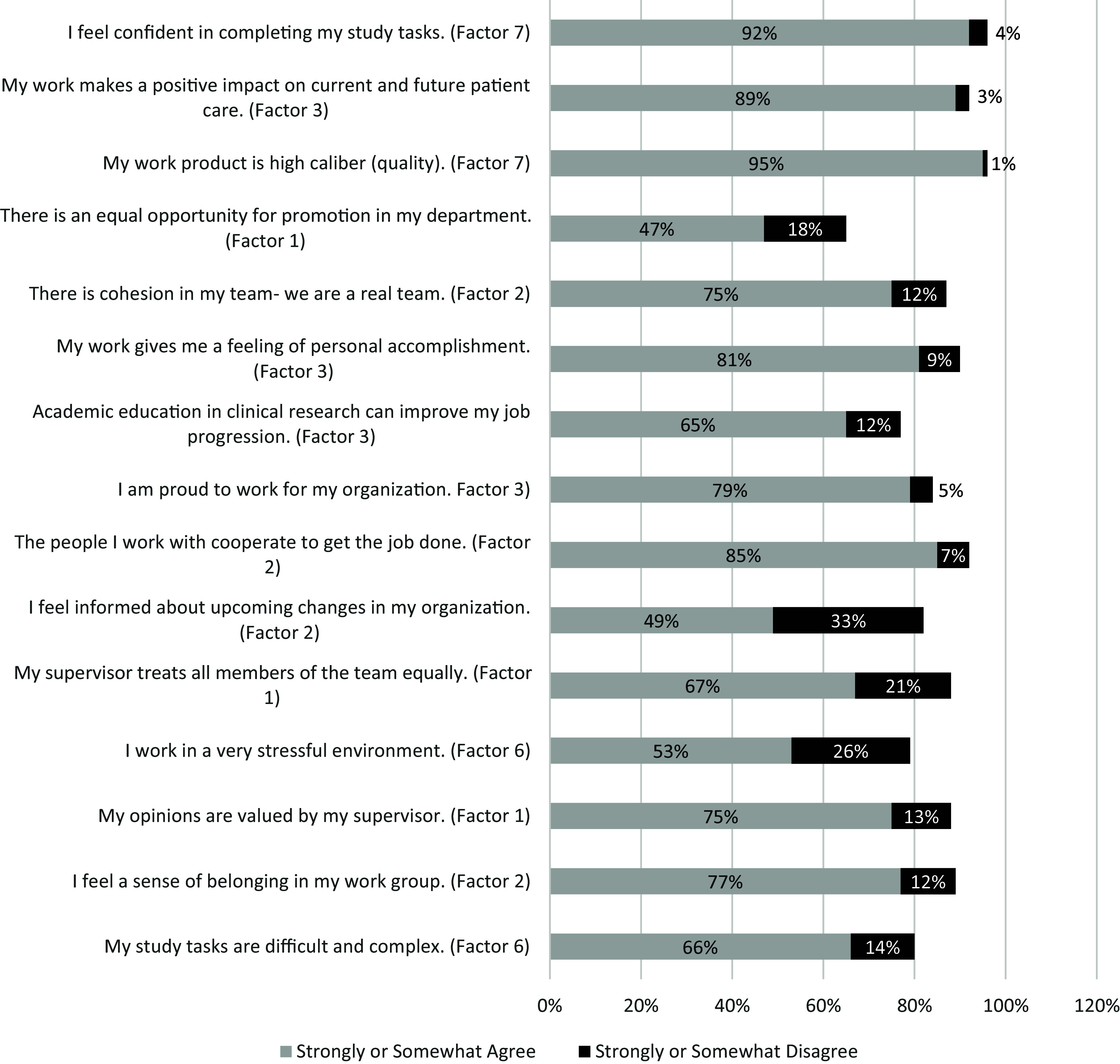
Results of “Indicate your level of agreement with the following statements:” (*N* = 484).

Analysis of responses to the 25 rating items in the final survey indicated the five most highly rated items across four factors included: 1) producing high-quality work (factor: work self-efficacy) (95% strongly or somewhat agreed), 2) working conditions (factor: work environment) (93% selected extremely or very important), 3) confidence in completing job tasks (factor: work self-efficacy) (92% strongly or somewhat agreed), 4) making a positive impact on patient care (factor: pride in work and advancement) (89% strongly or somewhat agreed), and 5) salary (factor: salary and benefits) (89% selected extremely or very important). Figures 1–3 provide a complete summary of quantitative data results, summarized by the survey’s three groups of questions. No Opinion, N/A, and Neither Agree nor Disagree responses were dropped for Figures 1–3.

### Qualitative results

Survey respondents provided 1,032 responses to the survey question: “Please list 3 things on your wish list that would make your role in clinical research more satisfying.” The qualitative content analysis confirmed all seven factors not only identified in the quantitative data but also identified one additional theme, which was the need for Training and Professional Development. Table 5 provides a complete summary of the content analysis results, including the number of instances each theme was reported and a respective percentage of frequency in the dataset. Themes are reported in order of frequency from highest to lowest, enabling easy identification of top priority areas.

**Table 5.** Results of qualitative content analysis of responses to item “Please list 3 things on your “wish list” that would make your role in clinical research more satisfying” (In order of highest-lowest frequency) (*N* = 1,032)

Factor/Theme n (%)^*^	Representative Quotations
Factor 6: Work Complexity and Stress 216 (21%)	“More diverse task list.” “The option to work from home or work four 10 hour shifts. There are numerous tasks that could be completed at home, where I could be more focused with less distractions.” “No weekend work” “Less sponsor-imposed paperwork and red tape.” “Reduced stigma around taking vacations” “Staff stabilization. Turnover has been frequent in my 12.5 years as a research professional, but over the last 2.5 years it has been excessive. Training and retraining is mentally exhausting for myself as well as team members.”
Factor 5: Salary and Benefits 198 (19%)	“A (higher) salary befitting my current volume of work and job performance.” “Incentives/Rewards for good work (e.g., promotion, higher salary, more vacation days - not necessarily all 3)” “Better salary-I can go work for a CRO for twice as much as I make now, but the job wouldn’t be as satisfying. However, money talks and doubling your salary is attractive to some people.”
Factor 1: Appreciation and Workplace Equity 195 (19%)	“Equal opportunity for career development and advancement” “Equal treatment amongst team members (no favoritism from investigators/managers).” “Appreciation from Leadership- In this environment, it is difficult to feel valued by the “leadership.” As a manager, I try to make my staff feel valued, but it is difficult when all their requests are answered with a “no.” Simple requests, such as supplies needed to do your job or flexible scheduling is a low priority to leadership and this makes staff members feel less valued.” “More departmental leadership recognition for my work” “Being acknowledged in more publications for the work that I do.”
Factor 4: Work Environment 162 (16%)	“Standardized processes, SOPs and working guidelines” “That there was an environment of mutual respect and kindness between research departments and coordinating departments such as investigators.” “Improved systems and reduced redundancy” “Easy to use tools and resources to quickly and accurately identify eligible patients for research studies” “Dedicated research space to see patients within my division” “Appropriate mechanisms and experienced human resources within institutional infrastructure to move administrative tasks in timely manner (MOUs, subawards, finance elements, etc.).” “Central workspace for my team. We are scattered.”
Training and Professional Development 105 (10%)	“More time for training and education. It is offered, but there is no time to dedicate to the training.” “Funding for certification in clinical research” “More professional development” “Opportunities for education/networking (e.g. conferences)” “More comprehensive training/educational resources”
Factor 2: Team 49 (5%)	“Increased teamwork and camaraderie amongst other subspecialty research teams within my department that I interact with on a day-to-day basis.” “A team, lack of management over the years has led to no cohesiveness among several on the team. which has led to no support. Coordinator back-up is strictly names on documents.“ “More inclusive team/less competitiveness”
Factor 7: Work Self-Efficacy 39 (4%)	“More input on the studies we choose to do” “Authority to compel teams to schedule quality reviews and respond to findings” “More active role in study decision making” “More involvement in decisions for our team.”
Factor 3: Pride in Work and Advancement 20 (2%)	“Value in research team as bright and competent individuals” “An environment that is more focused on better outcomes for study participants/patients than obtaining funding” “Advancement opportunities without having to leave a company.” “Clear opportunities/plans for promotion.”

* *n*=number of instances of the theme in the dataset and %=overall frequency of the theme.

## Discussion

Job satisfaction is a complex concept that is highly dependent on both extrinsic and intrinsic factors [22–24]. Understanding CRP job satisfaction and resolving related challenges is critical to addressing turnover rates in the research workforce at AMCs. Job satisfaction is not well-studied as a method to improve retention in this population, although other methods have been explored. Duke University reduced CRP turnover from 23 to 16% (a 30% improvement) after implementing a competency-based framework to better define job titles and a career ladder[29]. This approach supports several factors identified in this study: supervisor quality and workplace equity, work self-efficacy, work environment, salary and benefits, and work complexity and stress. Duke also developed on-demand onboarding training for CRPs as part of their competency-based workforce initiative, which may also impact several factors in this study: team, work self-efficacy, work complexity and stress, and education and professional development [30]. “Stay interviews” are also currently under study for their effectiveness in facilitating discussions between staff and managers to improve job satisfaction and retention through a multi-institutional initiative [31]. Stay interviews have particular promise for understanding and improving two key factors from this study: self-efficacy and pride in work. These two factors are inherently intrinsic, making them difficult to address with extrinsic changes like improving job ladders or offering higher salaries. Self-determination theory, a framework commonly used in organizational psychology, could also play a critical role in interpreting CRP job satisfaction [32,33].

We noted that the questions related to principal investigators (PIs) that were included in our original PCA did not have sufficient influence on any of the factors to be included in the final assessment tool. We believe that the relationships between CRPs and project PIs are important to job satisfaction either directly or through the way the overall project is organized. However, PI-related questions’ high correlation with questions about appreciation by supervisors and others were likely the cause of these items being excluded. This is an area that needs additional research, and possible refinements to the assessment tool should be explored.

This study has several limitations. The survey was administered at three unique AMCs who are actively addressing clinical research workforce challenges [34]. Given that many job satisfaction factors are heavily context-dependent, our study population might not reflect CRP experiences at other institutions. It will be important to use this instrument at other institutions to determine if the same factors identified in this study remain critical in other survey populations. Our survey respondents were primarily white females, limiting our ability to generalize results to a more diverse population, although race, gender, and salary levels in this study population are comparable to national data [35–37]. There are important qualitative findings that are difficult to measure via quantitative means (e.g., training and professional development). But training and professional development intersect multiple quantitative factors, making it important to administer the survey using mixed methods to better understand this complex issue. As future studies are being planned, we encourage the use of focus groups to inform item selection and design open-ended survey items.

## Conclusion

This study provides a comprehensive analysis of a CRP job satisfaction survey resulting in the development of a shorter (25 item) index to measure levels of job satisfaction across seven key factor domains. In a field characterized by high turnover, this newly developed CRP job satisfaction instrument may help identify workforce issues that impact retention of competent staff members, providing AMCs an opportunity to address the issues to improve retention and decrease turnover. The mixed method approach is essential to adequately describe and understand the complex factors that influence CRP job satisfaction.

## Supporting information

Knapke et al. supplementary material Knapke et al. supplementary material

## Supplementary material

The supplementary material for this article can be found at https://doi.org/10.1017/cts.2025.34.
