# Supplementary material for: Development of a job satisfaction measure for clinical research professionals: A mixed methods approach
Source: J Clin Transl Sci. 2025 Mar 24;9(1):e55. doi: 10.1017/cts.2025.34 (PMC11975783; doi:10.1017/cts.2025.34)
Supplement: Knapke et al. supplementary material [file S2059866125000342sup001.docx]

**Supplement 1: CRP Job Satisfaction Index**

| **Q1. How important to your job satisfaction are the following factors?** | Not at all important (1) | Slightly important (2) | Moderately important (3) | Very important (4) | Extremely important (5) | N/A or No Opinion (0) |
| --- | --- | --- | --- | --- | --- | --- |
| Salary (1) |  |  |  |  |  |  |
| Promotion Outlook (2) |  |  |  |  |  |  |
| Benefits (3) |  |  |  |  |  |  |
| Working Conditions (4) |  |  |  |  |  |  |
| Co-Workers (5) |  |  |  |  |  |  |
| The work itself (6) |  |  |  |  |  |  |

| **Q2. Please indicate how appreciated you feel by the following groups of people?** | Not at all appreciated (1) | Somewhat unappreciated (2) | Neutral (3) | Appreciated (4) | Extremely appreciated (5) | N/A (0) |
| --- | --- | --- | --- | --- | --- | --- |
| Your team (8) |  |  |  |  |  |  |
| Your department (9) |  |  |  |  |  |  |
| Your organization (institution) (10) |  |  |  |  |  |  |
| Your supervisor (11) |  |  |  |  |  |  |

| **Q3. Please indicate your level of agreement with each of the following statements.** | Strongly disagree (1) | | Somewhat disagree (2) | | Neither agree nor disagree (3) | | Somewhat agree (4) | | Strongly agree (5) | | N/A (0) |
| --- | --- | --- | --- | --- | --- | --- | --- | --- | --- | --- | --- |
| My study tasks are difficult and complex (12) |  | |  | |  | |  | |  | |  |
| I feel a sense of belonging in my work group (13) |  |  | |  | |  | |  | |  | |
| My opinions are valued by my supervisor (15) |  |  | |  | |  | |  | |  | |
| I work in a very stressful environment (16) |  |  | |  | |  | |  | |  | |
| My supervisor treats all members of the team equally (17) |  |  | |  | |  | |  | |  | |
| I feel informed about upcoming changes in my organization (18) |  |  | |  | |  | |  | |  | |
| The people I work with cooperate to get the job done (19) |  |  | |  | |  | |  | |  | |
| I am proud to work for my organization (20) |  |  | |  | |  | |  | |  | |
| Academic education in clinical research can improve my job progression (21) |  |  | |  | |  | |  | |  | |
| My work gives me a feeling of personal accomplishment (22) |  |  | |  | |  | |  | |  | |
| There is cohesion in my team- we are a real team (23) |  |  | |  | |  | |  | |  | |
| There is an equal opportunity for promotion in my department (24) |  |  | |  | |  | |  | |  | |
| My work product is of high caliber (quality) (25) |  |  | |  | |  | |  | |  | |
| My work makes a positive impact on current and future patient care (26) |  |  | |  | |  | |  | |  | |
| I feel confident in completing my study tasks (27) |  |  | |  | |  | |  | |  | |

**Q4. Please list three things on your “wish list” that would make your role in clinical research more satisfying:**
